# Pleomorphic adenoma arising from ectopic salivary glands in the submandibular region: a case report

**DOI:** 10.1093/jscr/rjag475

**Published:** 2026-06-19

**Authors:** Yunosuke Ikeda, Atsushi Ogawa, Tadashi Kawai, Shintaro Kogi, Mitsuru Izumisawa, Hiroyuki Yamada

**Affiliations:** Division of Oral and Maxillofacial Surgery, Department of Oral and Maxillofacial Reconstructive Surgery, School of Dentistry, Iwate Medical University Dental and Oral Surgery, 19-1 Uchimaru, Morioka, Iwate 020-8505, Japan; Dentistry and Oral Surgery, Iwate Prefectural Kuji Hospital, 32-3 Asahicho, Kuji, Iwate 028-8040, Japan; Mikinogahara Dental Clinic, Towada, Higashiichibanncho 10-1034-0012, Japan; Division of Oral and Maxillofacial Surgery, Department of Oral and Maxillofacial Reconstructive Surgery, School of Dentistry, Iwate Medical University Dental and Oral Surgery, 19-1 Uchimaru, Morioka, Iwate 020-8505, Japan; Division of Oral and Maxillofacial Surgery, Department of Oral and Maxillofacial Reconstructive Surgery, School of Dentistry, Iwate Medical University Dental and Oral Surgery, 19-1 Uchimaru, Morioka, Iwate 020-8505, Japan; Division of Oral Radiology, Department of Oral and Maxillofacial Reconstructive Surgery, School of Dentistry, Iwate Medical University, 19-1 Uchimaru, Morioka, Iwate 020-8505, Japan; Division of Oral and Maxillofacial Surgery, Department of Oral and Maxillofacial Reconstructive Surgery, School of Dentistry, Iwate Medical University Dental and Oral Surgery, 19-1 Uchimaru, Morioka, Iwate 020-8505, Japan

**Keywords:** pleomorphic adenoma, ectopic salivary gland tissue, submandibular region

## Abstract

Pleomorphic adenoma (PA) or mixed tumor, is the most common benign tumor of the major and minor salivary glands. However, PA arising from ectopic salivary gland tissue in the submandibular region is extremely rare with only four case reports described in the English language literature. An 80-year-old Japanese woman presented with a growing mass measuring 25 mm in diameter in the right submandibular region. Magnetic resonance imaging revealed a well-demarcated mass with low intensity on T1-weighted images and high intensity on T2-weighted images with heterogeneous signal intensity. The submandibular gland was compressed, but preserved fat plane was noted at the boundary between the tumor and the gland. The excised specimen was diagnosed as a PA arising from ectopic salivary gland tissue in the submandibular region. This case highlights the diagnostic difficulty of ectopic PA in the submandibular region and the importance of careful preoperative evaluation to avoid unnecessary extensive surgery.

## Introduction

Pleomorphic adenoma (PA) is the most common benign salivary gland tumor, accounting for 45.5% of all salivary gland tumor [1]. Ectopic salivary gland tissue may occur in the head and neck, including within lymph nodes, and can give rise to neoplasms. However, PA arising from ectopic salivary gland tissue in the submandibular region is extremely rare, with only four cases reported in the English literature to date [2–5]. These tumors lack anatomical continuity with the native salivary glands and may present as isolated submandibular masses, making differentiation from lymph node metastasis or primary submandibular gland tumors difficult on clinical and radiological grounds. Although fine-needle aspiration cytology (FNAC) is commonly used, its diagnostic accuracy may be limited in ectopic lesions.

This diagnostic uncertainty may lead to more extensive surgery, including unnecessary gland excision or lymph node dissection when malignancy cannot be excluded. The optimal preoperative diagnostic approach and surgical strategy remain unclear due to the rarity of this condition.

Herein, we report a rare case of ectopic PA in the submandibular region. This case highlights the diagnostic challenges and the importance of careful preoperative evaluation to avoid overtreatment.

## Case report

An 80-year-old Japanese woman presented with a growing mass in the right submandibular region. A painless mass had developed several years earlier and recently enlarged. She had a history of diabetes and osteoporosis. Examination revealed a firm, painless mass measuring 25 mm in diameter in the right submandibular region under the skin (Fig. 1). Ultrasonography revealed a well-circumscribed solid mass measuring approximately 23 × 18 × 17 mm near the right submandibular gland. Although the mass was adjacent to the submandibular gland, no beak sign was observed, suggesting the absence of pathological continuity between the gland and the mass (Fig. 2). Magnetic resonance imaging revealed a well-demarcated mass with low intensity on T1-weighted images and high intensity on T2-weighted images, with heterogeneous signal intensity. The right submandibular gland was compressed; however, an additional fat signal was observed at the boundary between the tumor and the submandibular gland, and no apparent continuity was noted (Fig. 3A–D). FNAC was not performed preoperatively because malignancy was strongly suspected and surgical excision was planned irrespective of cytological findings. Because malignancy could not be excluded, the tumor, surrounding lymph nodes, and submandibular gland were excised via submandibular triangle dissection (Fig. 4A). The specimen measured 30 × 16 × 16 mm, was encapsulated, firm, and separated from the gland (Fig. 4B). Pathological examination showed a well-circumscribed pleomorphic adenoma with duct-forming cells, spindle-shaped cells in myxoid/fibrous stroma, and chondroid differentiation. No atypia or mitoses were observed. The tumor was surrounded by fibrous and lymphoid layers (Fig. 5A and B), supporting its origin from ectopic salivary gland tissue within a lymph node. No histopathological features suggestive of malignancy or other salivary gland neoplasms were identified. These findings confirmed PA arising from ectopic salivary gland tissue in the submandibular region. The patient remained asymptomatic and recurrence-free at 20 months.

**Figure 1:**
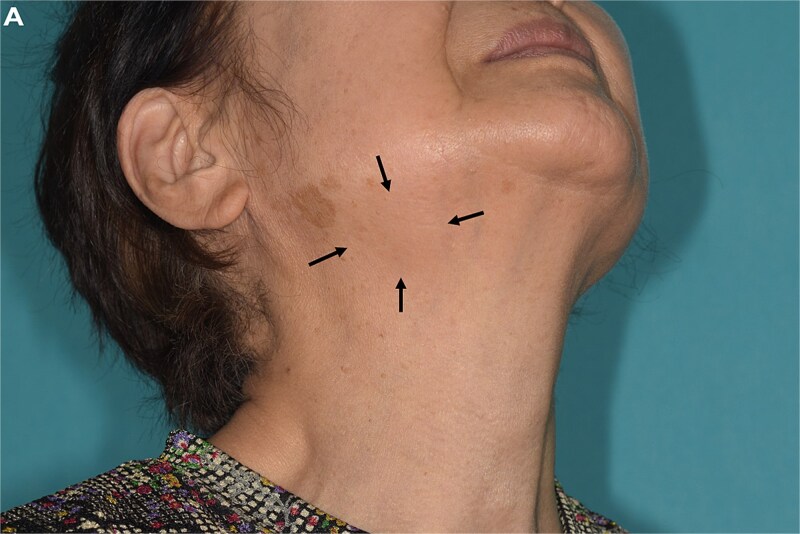
A 25-mm solitary mass (arrows) in the right submandibular region.

**Figure 2:**
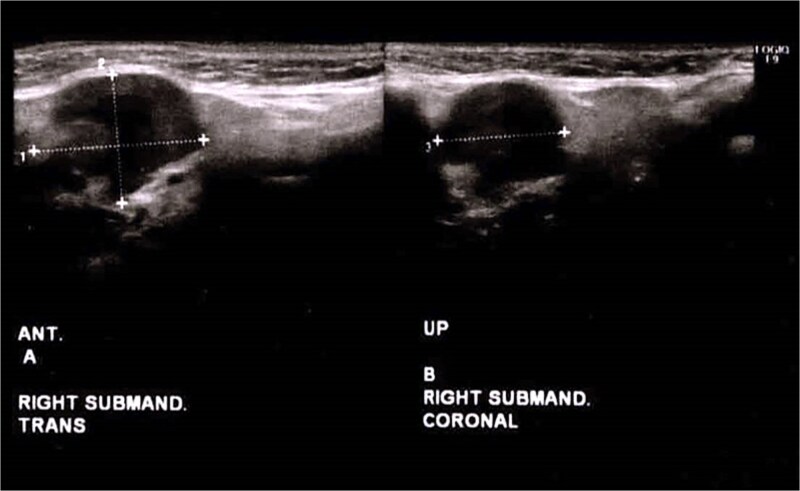
Ultrasonography reveals a well-circumscribed solid mass of approximately 23 × 18 × 17 mm near the right submandibular gland. Although it is attached to the submandibular gland, no beak sign is observed, thus suggesting that the mass developed outside the gland.

**Figure 3:**
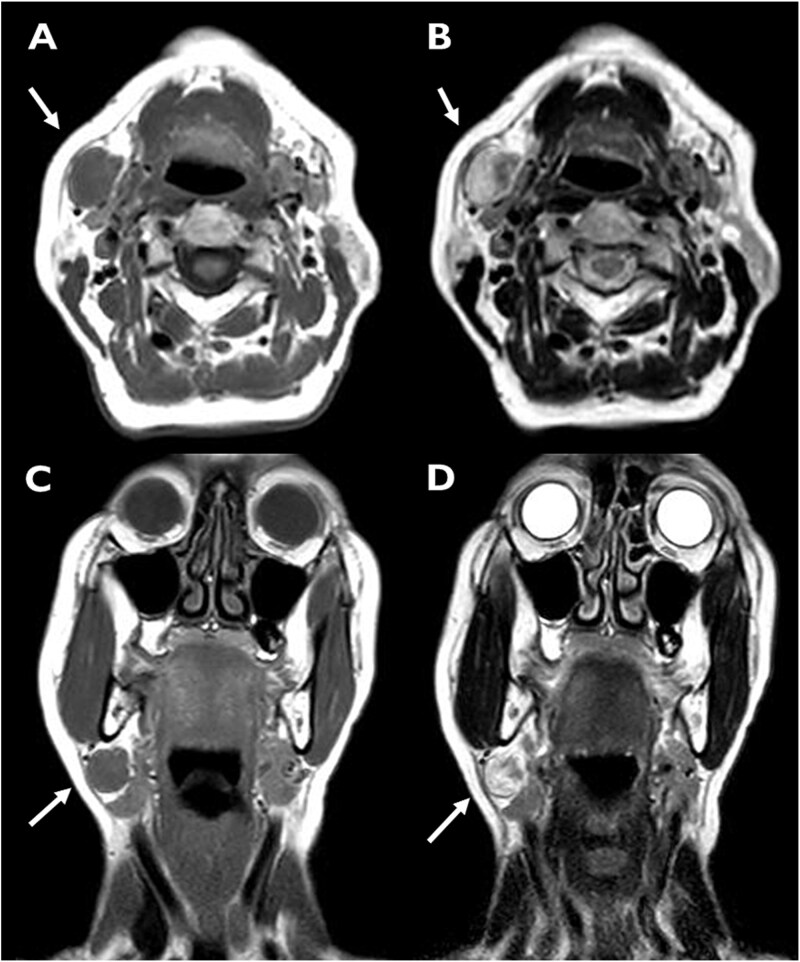
MRI showing a well-defined mass in the right submandibular region. The lesion demonstrates low signal intensity on T1-weighted images (A) and heterogeneous high signal intensity on T2-weighted images (B). A preserved fat plane is observed between the tumor and the submandibular gland, indicating no continuity (C, D).

**Figure 4:**
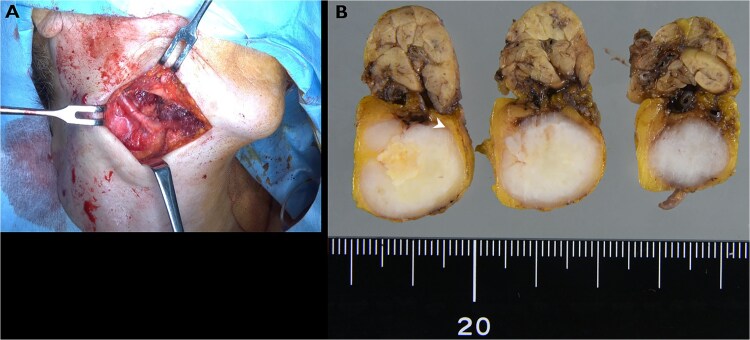
Surgical and specimen findings. The tumor was excised en bloc with the submandibular gland and adjacent tissue via submandibular triangle dissection (A). The resected specimen shows a well-encapsulated tumor separated from the gland (B).

**Figure 5:**
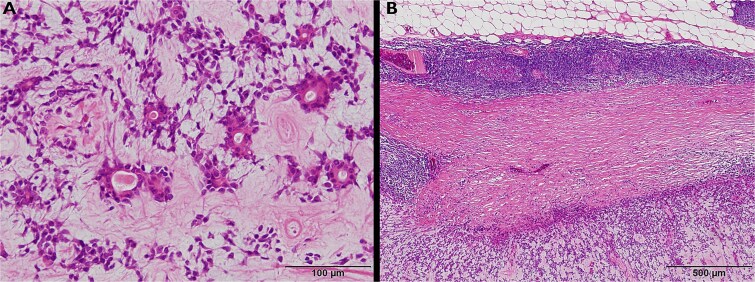
Histopathological findings. The tumor shows ductal and spindle cell components in a myxoid and fibrous stroma (A, hematoxylin and eosin stain, ×200). The capsule is composed of fibrous and lymphoid tissue (B, ×40).

## Discussion

Ectopic salivary gland tissue may arise in various head and neck locations, including lymph nodes [6]. Bernier *et al*. [7] and Cotelingam *et al*. [8] reported that some para-parotid and cervical lymph nodes contain salivary glands. The tissue of origin was thought to be an ectopic salivary gland that had invaded the submandibular lymph nodes.

PA typically arises in salivary glands and is less commonly found in ectopic locations. Thus, PAs have been previously described in the external auditory canal, skin, breast tissue, and vulva and account for half of all lacrimal gland tumors [9]. However, in the last 45 years, only four patients who developed PA in the ectopic salivary gland tissue in the submandibular region have been reported [2–5] (Table 1). Reported cases, showed favorable outcomes after surgery. The tumor size ranged from 15 to 25 mm. Resection was selected as the treatment choice in all patients, and recurrence was not reported in any patient. The patient in our case report showed a pathologically typical benign PA encapsulated by thick, dense fibrous, and lymphoid tissues.

**Table 1:** Reported cases of pleomorphic adenoma arising from the ectopic salivary glands in the submandibular region.

No	Author	Year of publication	Age	Gender	Size (mm)	Treatment	Follow-up (months)	Recurrence
1	Zajtchuk *et al.* [2]	1982	30	F	Unable to obtain	Extirpation of mass	Unable to obtain	no
2	Ohsawa *et al.* [3]	2002	60	F	15	Extirpation of mass	Unable to obtain	no
3	Kubota *et al.* [4]	2005	61	M	25×25	Extirpation of mass	84	no
4	Iwai *et al.* [5]	2021	55	M	20×20	Extirpation of mass	84	no
5	Present case	2022	80	F	25	Extirpation of mass+submandibular grand excision	11	no

Based on the clinical presentation and localization of the lesion, the differential diagnosis in this case included other benign or malignant salivary glands and metastatic tumors. PA consists of epithelial and myoepithelial elements within a myxochondroid stroma [10]. A complete or an incomplete capsule is typically formed by the surrounding salivary parenchyma [10]. The specimen excised from our patient revealed a solitary, soft, well-circumscribed, encapsulated nodule. Clinical determination of whether the tumor was benign or malignant was difficult before surgery. In this case, preservation of the fat plane and absence of a beak sign on magnetic resonance imaging (MRI) suggested lack of continuity with the submandibular gland, however, malignancy including metastatic lymph node or primary salivary gland tumor, could not be excluded despite imaging findings. Therefore, en bloc excision including the submandibular gland and adjacent lymph nodes was performed for definitive diagnosis and complete resection.

The definitive treatment for PA is surgery, and the type of procedure varies depending on the patient’s age, tumor size, and tumor location. Although these tumors are encapsulated, resection with adequate margins is essential to avoid recurrence [10]. Previous reports have suggested a potentially higher risk of malignant transformation and metastasis in ectopic salivary gland tumors, although the evidence is limited [11, 12]. Therefore, resection of the tumor, including the surrounding tissue and lymph nodes, was considered necessary in this case. Similarly, the en bloc resection of the tumor and associated glands is the treatment of choice for PAs of the submandibular gland [13]. Recurrence occurs in 4% of patients with PAs originating from the salivary gland, with those most at risk of recurrence having irregular borders, thus increasing the likelihood of positive margins [9]. The average time for recurrence was 7–10 years [9]. Malignant transformation is rare but increases with long-standing tumors [14]. The follow-up period in this case is relatively short (20 months), and careful long-term follow-up is warranted, as recurrence of pleomorphic adenoma has been reported to occur 7–10 years after treatment [15]. Therefore, early definitive treatment and longer-term follow-up of patients are strongly recommended.
